# Fortasyn Connect Improves Neuropsychiatric Symptoms in Patients with Mild Cognitive Impairment and Dementia: Results from a Retrospective Real-World Study

**DOI:** 10.3233/JAD-221122

**Published:** 2023-05-16

**Authors:** Miquel Aguilar-Barberà, Paquita Soler-Girabau, Ana Isabel Tabuenca-Martín, Laura Prieto-del Val

**Affiliations:** a Institut de Neurologia i Neuropsicologia de Sabadell, Barcelona, Spain; b Dynamic Science (Evidence Clinical Research), Madrid, Spain

**Keywords:** Alzheimer’s disease, anxiety, apathy, dementia, depression, mild cognitive impartment, neuropsychiatric symptoms

## Abstract

**Background:**

Behavioral and psychological symptoms of dementia (BPSD) manifest in the early stages of the disease and impair patients’ and caregivers’ quality of life.

**Objective:**

To assess the effectiveness of the nutritional supplement Fortasyn Connect on BPSD for 12 months in people with mild cognitive impairment (MCI) and dementia in clinical practice.

**Methods:**

Retrospective, national, single-center study of 236 patients (158 MCI and 78 dementia; 55.1% of AD etiology). BPSD were assessed with the Neuropsychiatric Inventory (NPI) at month 3, 6, and 12. Cognition (Mini-Mental State Examination, MMSE), depression (Geriatric Depression Scale, GDS), and everyday functioning (Blessed Dementia Scale, BLS-D; Rapid Disability Rating Scale 2, RDRS2) were also evaluated.

**Results:**

Total NPI score, caregiver impact, and symptoms of depression, anxiety, apathy, and irritability improved after 3, 6, and 12 months from Fortasyn Connect initiation (*p* < 0.001). NPI decreases were more pronounced when baseline NPI score was higher than > 20 points (*p* < 0.001). The benefit was independent of gender, age, diagnosis, etiology, or concomitant treatment (*p* < 0.0001), although larger decreases in NPI total score were observed in MCI patients (*p* < 0.0001). After 12 months, GDS scores decreased (*p* = 0.042), and MMSE, BLS-D, and RDRS 2 scores remained stable.

**Conclusion:**

Fortasyn Connect improved BPSD over at least a year in patients with MCI and dementia. Depression, anxiety, apathy, and irritability were the symptoms that improved the most. The benefit was independent of patients’ characteristics and treatment but was greater if prescribed early and when baseline NPI scores were higher.

## INTRODUCTION

Around 55 million people have dementia worldwide, with Alzheimer’s disease (AD) as the most common cause. The number of these patients is expected to rise up to 139 million in 2050 [1], mainly due to population ageing and growth [2]. Long before the diagnosis of dementia, patients present a symptomatic stage known as mild cognitive impairment (MCI) [3]). At this stage, patients not only suffer cognitive deficits, but also behavioral and psychological symptoms of dementia (BPSD), also known as neuropsychiatric symptoms [4, 5], including depression, anxiety, and apathy [6, 7]. As a result, MCI already imposes a substantial disease burden [8] and has a negative impact on patients’ [9] and caregivers’ [10] quality of life.

One of the pathological changes observed in patients with dementia and MCI is synaptic loss [11–13]. Synaptic disruption contributes to cognitive and neuropsychiatric symptoms and might be a downstream effect of amyloidosis, tauopathy, inflammation, and other pathological processes [14]. In the European context, where no disease-modifying therapies are currently approved, the role of symptomatic treatments (cholinesterase inhibitors or NMDA receptor antagonists) is key for delaying cognition and neuropsychiatric symptoms, especially when introduced early on in the disease stage [15, 16]. Moreover, nutritional interventions have attracted great interest since they are part of the lifestyle factors that can be modified to delay cognitive impairment and strengthen brain functioning [17]. Nutritional interventions play a role in counteracting synaptic loss and reducing membrane-related pathology in AD, by providing nutritional precursors and cofactors that support neuronal membrane formation and function [18].

Souvenaid is a nutritional supplement that contains Fortasyn Connect, a multi-nutrient combination (B vitamins, vitamin C, vitamin E, long-chain omega-3 fatty acids, choline, uridine, and selenium) that supports synapse formation and function. The LipiDiDiet randomized, double-blind, placebo-controlled clinical trial showed a significant benefit over a treatment period of 3 years in cognition, function, brain atrophy, and disease progression in people with prodromal AD [19]. However, the effect of Fortasyn Connect on BPDS remains unexplored. Only one real-world study published so far has assessed BPSD symptoms in patients with MCI and AD, showing improvements especially in MCI patients [20]. However, this study did not use a validated tool to assess BPSD. In the present study, we evaluated the effectiveness of Fortasyn Connect during 12 months on BPSD using the Neuropsychiatric Inventory (NPI) in people with MCI and dementia in real-world clinical practice. The effect of Fortasyn Connect on cognition and function was also assessed.

## METHODS

### Study design and patients

This was a retrospective, national, single-center real-world study designed to assess the effectiveness of Fortasyn Connect in the clinical setting. The study was conducted at the Institut de Neurologia i Neuropsicologia of Sabadell (Barcelona). Patients were included in the study if they met the following selection criteria: a diagnosis of MCI or dementia (AD, Parkinsonism–dementia complex [dementia with Lewy bodies, Parkinson’s disease dementia, Parkinson plus], frontotemporal dementia, vascular dementia, or secondary dementia), were≥50 years old, and had received Fortasyn Connect during at least 3 months from May 13, 2013 to June 30, 2021. Patients who were participating in a clinical trial were excluded.

The diagnose of MCI or dementia was based on clinical criteria and confirmed by radiological and neuropsychological data according to routine clinical practice. The Global Deterioration Scale (GDS) was used to identify the presence and severity of dementia (score 3 corresponding to MCI, score 4 to mild dementia, score 5 to moderate dementia, score 6 to moderately severe dementia, and score 7 to severe dementia). The diagnosis of MCI was further confirmed by the DSM-V criteria: i) self- or informant-reported cognitive complaint, ii) objective cognitive impairment, iii) preserved independence in functional abilities, iv) no dementia. The diagnosis of AD was established according to NINCS-ADRDA criteria [21]. The etiology for dementia or MCI was determined by neuropsychology tests, neuroimaging (computed tomography, magnetic resonance imaging [MRI], amyloid- Positron Emission Tomography [PET], ^18^F-fluorodeoxyglucose [^18^F-FDG] PET, DatSCAN), and cerebrospinal fluid biomarkers (tau, phosphorylated tau, amyloid beta 1–40 and 1–42) when available. MCI patients with no conclusive results or absence of biomarkers were classified as MCI of unknown etiology.

The study was approved by the Ethics Committee of the Clínico San Carlos Hospital (Madrid) and complied with the Declaration of Helsinki and applicable local and European legislation on nutritional products and personal data protection. Due to the retrospective design of the study and according to the applicable regulations, a waiver of informed consent was granted for this study by the Ethics Committee.

### Assessments

BPSD were assessed by the NPI [22], which is instrument based on an interview of the caregivers. It evaluates 12 symptoms (delusions, hallucinations, agitation, depression, anxiety, euphoria, apathy, disinhibition, irritability, psychomotor alterations, sleep change, and eating change), for which the frequency is rated 1–4 and the severity is rated 1–3 (with higher numbers indicating greater frequency and severity) to obtain a total score for each symptom by multiplying the frequency by the severity. The NPI also assesses caregiver distress with scores ranging from 0 to 5 (0 = no distress; 5 = extreme distress)

Depression was evaluated by the Geriatric Depression Scale developed by Yesavage [23], a 30-item yes/no self-report questionnaire completed by the patient that is widely used to screen for depression in the elderly; scores≥11 suggests depression. Global cognitive function was assessed using the Spanish version of the Mini-Mental State Examination (MMSE) [24]. Everyday function was assessed by 1) the two first subscales of the Blessed Dementia Scale (BLS-D), which measures changes in performance of everyday activities (8 items) and changes in habits (3 items) and has a range from 0 (normal) to 17 (severely demented) [25], and by 2) two of the Rapid Disability Rating Scale 2 (RDRS2) subscales (RDRS2-I activities of daily living [ADL] and RDRS2-II disability); scores range from 8 to 32 (RDRS2-I) and from 7 to 28 (RDRS2-II) points. In the BLS-D and RDRS-2, higher scores indicate higher degree of impairment.

Assessments were conducted during routine visits at Fortasyn Connect initiation (baseline), usually after 3, 6, and 12 months. The frequency of visits depended on the monitoring needs of each patient, so some patients did not have a follow-up visit and were not assessed at month 3. Adverse events and reasons for treatment discontinuation were also collected at every study visit. At the visits, patients were asked by their neurologist to report if they had suffered any adverse event or lack of tolerability.

### Statistical analyses

We calculated that a sample size of 220 patients would be sufficient to detect a change in the NPI total score of 3.42 points over 12 months with a 5% alpha and a 20% beta error (a 5% data loss was assumed).

Quantitative data were presented as mean and standard deviation (SD) when normally distributed and as median and interquartile range (IQR) when skewed. Qualitative data were expressed as number of cases and valid percentages.

Quantitative variables were described using measures of central tendency and dispersion (mean and SD or median and IQR values). Qualitative variables were described using absolute and relative frequencies.

To compare data from paired samples (i.e., changes in NPI, GDS, BLS-D and RDR2 scores from one visit to another) Wilcoxon tests were used.

General linear models (GLM) of repeated measures were also conducted to detect changes (in NPI scores) including patients with available data at all time points (baseline, month 3, month 6, and month 12). GLM of repeated measures were also used for *post-hoc* analyses to evaluate the change in NPI scores according to baseline NPI scores (≤20 or > 20) in patients with available data at baseline, month 6, and month 12, where Hotelling’s trace was used.

A linear mixed model of repeated measures was conducted to analyze NPI total score changes over time (within subject factor) from baseline to each of the following visits considering several categorial variables (between subject factors: sex [male/female], age at Fortasyn Connect initiation [(< 71/71–80/> 80)], acetylcholinesterase inhibitors [AChEi, yes/no], antidepressants [yes/no], ginkgo biloba [yes/no], etiology [AD/other], grouped etiology and AD/PD/MCI of unknown etiology], and diagnosis [MCI/dementia). The Tukey multiple comparison test was used. All linear mixed models were calculated in the total sample size (*n* = 236) except for the grouped etiology where 214 patients were included.

All tests were two-tailed with a 5% significance level. Statistical analyses were performed using the Statistical Package for Social Sciences (version 28.0, SPSS Inc., Chicago, IL, USA).

## RESULTS

### Baseline characteristics

A total of 236 patients met all the selection criteria and were included in the study. Baseline characteristics of these patients are summarized in Table 1. Mean age was 76.1 (min-max, 58–90) years, and 58.1% were female. Mean (SD) MMSE score was 24.0 (3.9). Most patients had a diagnosis of MCI (66.9%) and the probable etiology was considered to be AD in 55.1% of the study population. Slightly more than half (56.4%) of patients were treated with AChEi and 22% received antidepressants. After 12 months, 35 (14.8%) patients discontinued receiving Fortasyn Connect. Main reasons for discontinuation were the price of the product (*n* = 14), progression of dementia (*n* = 4), adverse events (*n* = 4; diarrhea, 2; nausea, 1; abdominal discomfort, 1), lack of adherence (*n* = 3), exitus (*n* = 2), or other (*n* = 8). The decision to discontinue was made by the patients’ family (*n* = 18), treating physician (*n* = 10), patient (*n* = 3), other clinician (*n* = 2), or due to exitus (*n* = 2).

**Table 1:** Baseline characteristics at Fortasyn Connect initiation

Variable	Patients = 236
Sex (female), n (%)	137 (58.1)
Age (y), mean (SD)	76.1 (6.7)
Education (y), mean (SD)	8.1 (4.7)
MMSE, mean (SD)	24.0 (3.9)
Stage (GDS), n (%)
GDS < 3	1 (0.4)
GDS = 3	152 (64.4)
GDS≥4	83 (35.2)
Diagnosis
MCI	158 (66.9)
Dementia	78 (33.1)
Etiology, n (%)
AD	130 (55.1)
Parkinson dementia complex	38 (16.1)
Other (secondary^b^, vascular, frontotemporal)	22 (9.3)
MCI of unknown etiology	46 (19.5)
Treatment (yes), n (%)
AChEi	133 (56.4)^b^
Antidepressants	52 (22)
Antipsychotics	24 (10.2)
Anxiolytics	24 (10.2)
Ginkgo Biloba	28 (11.9)
Levodopa	31 (13.1)

^a^Secondary dementias: hydrocephalus and Wernicke -Korsakoff syndrome. ^b^The number of patients treated with AChEi was higher than the number of patients with dementia because some patients with MCI were also treated with AChEi.

### Changes in neuropsychiatric symptoms

Mean (SD) NPI scores (number of items, severity, caregiver distress, and total) significantly decreased after 3 months of Fortasyn Connect initiation and were sustained until month 12 (*p* < 0.001) (Table 2). Significant improvements in symptoms of depression, anxiety, apathy (*p* < 0.001), and irritability (*p* < 0.008) were observed at each visit (from month 3 to month 12). Significant improvements in delusions, hallucinations (*p* = 0.01), and sleep disorders (*p* = 0.038) were only observed at month 3, and in disinhibition at month 6 (*p* = 0.003) and month 12 (*p* = 0.01) (Table 2). At month 12, most patients (65.3%) showed improvements in the NPI (total NPI score≤–2), 12.2% patients remained stable (total NPI score + 1/–1), and 22.4% worsened (total NPI score≥2). The significant decreases in NPI scores were also observed when patients with MCI and dementia were considered separately (see Supplementary Tables 1 and 2). Decreases in depression, anxiety, apathy, and irritability were statistically significant in the MCI group at all visits (month 3, 6, and 12) (Supplementary Table 1). Decreases in these items were also observed in patients with dementia, although they were not statistically significant at all visits (Supplementary Table 2).

**Table 2:** NIP scores (all patients all visits)

NPI scores	Baseline (*n* = 236)^1^	Month 3 (*n* = 120)		Month 6 (*n* = 157)		Month 12 (*n* = 147)^1^	
	Mean (SD)	Mean (SD)	*p*	Mean (SD)	*p*	Mean (SD)	*p*
Number of items	3.7 (2.2)	2.5 (2.1)	< 0.001^*^	2.5 (2.0)	0.001^*^	2.5 (2.1)	< 0.001^*^
Severity	5.2 (3.7)	3.2 (3.3)	< 0.001^*^	3.2 (3.0)	< 0.001^*^	3.3 (3.4)	< 0.001^*^
Caregiver distress	10.2 (6.9)	5.7 (5.9)	< 0.001^*^	6.2 (6.1)	< 0.001^*^	6.5 (6.5)	< 0.001^*^
Total (severity and frequency)	17.9 (13.4)	10.4 (11.1)	< 0.001^*^	10.4 (11.2)	< 0.001^*^	10.8 (12.1)	< 0.001^*^
*Per item*						
Delusions	1.2 (2.6)	0.7 (2.0)	0,018^*^	0.8 (2.3)	0.294	0.8 (2.2)	0.070
Hallucinations	0.5 (1.6)	0.2 (0.9)	0.015^*^	0.3 (1.4)	0.105	0.3 (1.2)	0.562
Agitation	0.6 (1.7)	0.3 (1.3)	0.083	0.2 (1.2)	0.088	0.4 (1.5)	0.066
Depression	2.7 (3.2)	1.3 (2.4)	< 0.001^*^	1.5 (2.6)	< 0.001^*^	1.6 (2.7)	< 0.001^*^
Anxiety	3.1 (3.4)	1.5 (2.4)	< 0.001^*^	1.6 (2.5)	< 0.001^*^	1.4 (2.4)	< 0.001^*^
Euphoria	0.2 (1.0)	0.2 (1.0)	0.671	0.1 (0.7)	0.281	0.1 (0.8)	0.531
Apathy	3.8 (3.2)	2.6 (3.2)	< 0.001^*^	2.5 (2.9)	< 0.001^*^	2.5 (3.0)	< 0.001^*^
Disinhibition	0.5 (1.4)	0.3 (1.3)	0.394	0.1 (0.5)	0.003^*^	0.2 (1.0)	0.010^*^
Irritability	2.0 (2.5)	1.3 (2.0)	0.002^*^	1.0 (1.7)	< 0.001^*^	1.4 (2.2)	0.008^*^
Psychomotor alterations	0.7 (2.1)	0.3 (1.2)	0.162	0.3 (1.3)	0.091	0.3 (1.6)	0.039
Sleep disorders	1.5 (2.5)	0.7 (1.6)	0.038^*^	1.0 (1.9)	0.123	1.1 (2.0)	0.113
Eating disorders	1.1 (2.0)	1.0 (1.9)	0.389	0.9 (1.9)	0.809	0.7 (1.7)	0.028^*^

^1^Data per item was missing in one patient; *p* values from comparisons between each month (month 3, month 6, and month 12) versus baseline are showed (Wilcoxon test).

In those patients with available NPI assessment at all visits (*n* = 47), the GLM of repeated measures showed significant decreases in total NPI scores at month 3, month 6, and month 12 compared to baseline (*p* < 0.001).

**Fig. 1:**
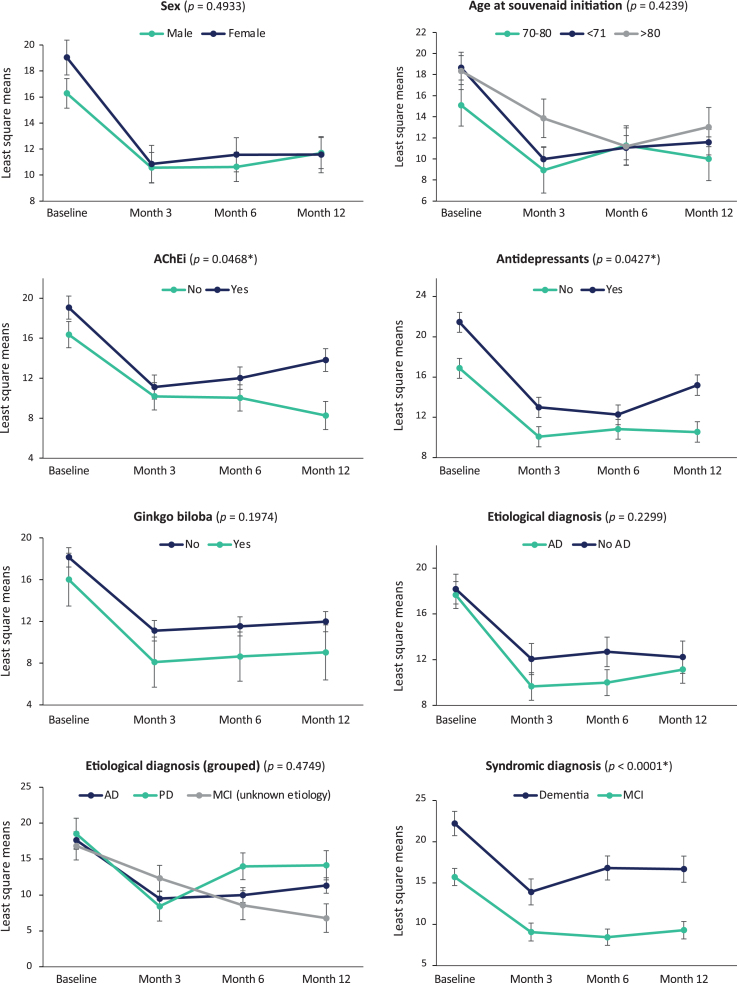
NPI total score changes per patient characteristics and treatment. Data are mean change from baseline as estimated by the mixed model.

The mixed model analysis (Fig. 1) showed that decreases in the NPI total score after Fortasyn Connect initiation (*p* < 0.0001) occurred regardless of sex, age, AChEi treatment, ginkgo biloba treatment, antidepressants, and etiologic or syndromic diagnosis. Nevertheless, more pronounced changes in NPI total score were associated with AChEi treatment (*p* = 0.0468), antidepressants (*p* = 0.0427), and diagnosis (*p* < 0.0001), with patients not receiving AChEi or antidepressants and those with MCI having even lower NPI total scores, especially at month 12.

**Fig. 2:**
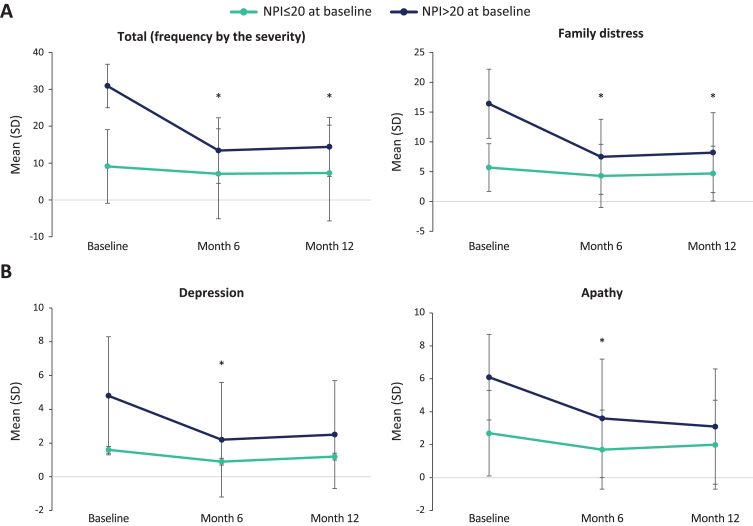
NPI score changes per baseline NPI total score (≤20, > 20) Only patients with data at each visit (baseline, month 6, and month 12) were included in the analyses; Significant comparisons between month 6 and month 12 versus baseline are shown.

*Post-hoc* analyses showed that patients with higher NPI total score at baseline benefited more from Fortasyn Connect. That is, patients with a baseline NPI total score > 20 had statistically significant decreases in the NPI total score at month 6 and month 12 from baseline (*p* < 0.001; *n* = 36), whereas these decreases were not significant in patients with a baseline NPI total score≤20 (*p* = 0.127; *n* = 75) (Fig. 2a). The same trend was observed between these subgroups in the change in other NPI scores: number of items (> 20, *p* < 0.001; ≤20, *p* = 0.102), severity (> 20, *p* < 0.001; ≤20, *p* = 0.072), caregiver distress (> 20, *p* < 0.001; ≤20, *p* = 0.076), delusions (> 20, *p* < 0.028; ≤20, *p* = 0.491), hallucinations (> 20, *p* < 0.032; ≤20, *p* = 0.840), agitation (> 20, *p* < 0.046; ≤20, *p* = 0.359), anxiety (> 20, *p* < 0.001; ≤20, *p* = 0.053), disinhibition (> 20, *p* < 0.021; ≤20, *p* = 0.373), irritability (> 20, *p* < 0.005; ≤20, *p* = 0.239), and psychomotor alterations (> 20, *p* < 0.034; ≤20, *p* = 0.726). On the other hand, decreases were significant in both groups in depression (> 20, *p* < 0.001; ≤20, *p* = 0.029) and apathy (> 20, *p* < 0.001; ≤20, *p* = 0.012) (Fig. 2b), and were not significant in euphoria (> 20, *p* < 0.241; ≤20, *p* = 0.373), sleep disorders (> 20, *p* < 0.114; ≤20, *p* = 0.566) or eating disorders (> 20, *p* < 0.270; ≤20, *p* = 0.738) (for detailed information on the mean (SD) at each visit, see Supplementary Table 1).

### Changes in cognition, depression, and everyday function

Table 3 displays mean (SD) scores in the MMSE, GDS, BLS-D, and RDRS-2 from baseline to month 12, including comparisons between scores at each visit versus baseline. These results are also presented in patients with MCI (Supplementary Table 3) and patients with dementia (Supplementary Table 4), separately.

**Table 3:** MMSE, GDS, BLS-D, and RDRS-2 at each visit (*all patients all visits*)

Scores	Baseline	Month 3		Month 6		Month 12	
	Mean (SD) [n]	Mean (SD) [n]	*p*	Mean (SD) [n]	*p*	Mean (SD) [n]	*p*
MMSE	24.0 (3.9) [236]	24.4 (3.8) [125]	< 0.001^*^	24.6 (3.8) [161]	0.175	23.9 (4.5) [151]	0.554
GDS	9.4 (5.9) [210]	7.9 (5.8) [50]	< 0.001^*^	8.2 (5.3) [52]	0.006^*^	7.9 (5.0) [63]	0.042^*^
BLS-D	4.8 (3.2) [230]	3.7 (3.4) [118]	< 0.001^*^	3.6 (3.1) [149]	< 0.001^*^	4.2 (3.5) [139]	0.080
ADL	1.7 (1.3) [230]	1.3 (1.3) [118]	< 0.001^*^	1.4 (1.2) [149]	0.013^*^	1.7 (1.5) [139]	0.559
Habits	0.7 (1.1) [230]	0.7 (1.2) [118]	0.809	0.6 (1.1) [149]	0.866	0.7 (1.2) [139]	0.02^*^
RDRS-2	22.8 (5.0) [235]	22.4 (5.6) [120]	0.002^*^	22.2 (4.9) [155]	0.144	23.5 (6.0) [144]	0.094
ADL	9.8 (3.2) [235]	10.1 (3.8) [120]	0.205	9.7 (3.2) [155]	0.946	10.5 (4.0) [144]	0.007^*^
Disability	9.1 (1.8) [235]	8.9 (1.8) [120]	0.003^*^	8.9 (1.9) [155]	0.184	9.3 (2.2) [144]	0.184

*p* values from paired comparisons between each month (3, 6, and 12) versus baseline are showed (Wilcoxon test). Comparisons were conducted considering the sample of patients with available data at each month and the same patients at baseline. Baseline scores for all patients with available data at each visit are showed. Baseline scores corresponding to the specific sample with available data at each following visit are, therefore, not showed.

MMSE total score significantly decreased at month 3 (mean [SD] change versus baseline: 0.8 [2.6]; *p* < 0.001) and remained stable until month 12 (mean [SD] change versus baseline: –0.1 [3.1]; *p* = 0.554). The percentage of patients with improvements in the MMSE total score was 49.6% at month 3, 44.1% at month 6, and 35.8% at month 12. GDS scores were significantly lower while receiving Fortasyn Connect at month 3 (*p* < 0.001), month 6 (*p* = 0.006), and month 12 (*p* = 0.042). The percentage of patients with depression (≥11 points in the GDS) decreased throughout this period (from 39% at baseline to 30.2% at month 3, 33.4% at month 6, and 29.2% at month 12). BLS-D scores also significantly declined up to month 6 (*p* < 0.001) but did not reach statistical significance at month 12 (*p* = 0.080). Almost half of patients (49.2%) showed improvements in the BLS-D scores at month 3, and slightly more than half of patients at month 6 (55.7%), and at month 12 (53.2%). When the two BLS-D scales were considered, scores in the ADL subscale were lower at month 3 (*p* > 0.001) and 6 (*p* = 0.013) but not at month 12 (*p* = 0.559), and scores in the habits subscale were higher at month 12 (0.5 [0.8] at baseline versus 0.7 [1.2] at month 12; *p* = 0.020). Scores in the ADL subscale of the RDRS-2 were sustained up to month 6 and increased at month 12 (*p* = 0.007). Scores in the disability subscale decreased at month 3 (*p* = 0.003) and did not show changes in the rest of visits.

## DISCUSSION

The present study has shown, for the first time, that Fortasyn Connect improves BPSD (especially symptoms of depression, anxiety, apathy, and irritability) during at least 12 months in patients with MCI or dementia. The benefit of Fortasyn Connect was independent of patient’s sociodemographic (sex, age) and clinical (etiology, diagnosis) characteristics, and whether they were receiving symptomatic treatments (AChEi, antidepressants, ginkgo biloba). Also, patients with higher total NPI scores at baseline showed larger reductions in BPSD compared to patients with lower baseline scores. Over this period, cognitive and everyday function remained stable. Among the main strengths of the present study are the large and well-characterized sample of real-world patients, the use of the same validated neuropsychological tests at each visit, and the assessment of several aspects affected at the early phases of dementia (cognitive status, function, depression, and other neuropsychiatric symptoms) in a real clinical setting.

Around 40% of worldwide dementias are attributed to modifiable risk factors, including nutrition, suggesting that the delay or prevention of these cases may be possible [26]. Accordingly, the guideline on MCI management from the American Academy of Neurology (AAN), encourages clinicians to first treat modifiable risk factors that may be contributing to cognitive impairment in patients diagnosed with MCI [27]. Single nutritional supplements have limited evidence of clinical benefit, but multi-nutrient interventions have revealed promising results in patients with MCI and dementia [17, 28] and are therefore recommended by the AAN [27]. The benefits of combining multimodal lifestyle interventions could be mediated, at least in part, by normalization in the phospholipid metabolism, which is altered in MCI and dementia patients [29]. By increasing levels of the nutrients needed for phospholipid synthesis, dietary interventions may contribute to improving synaptic formation and function while also preserving neuronal activity in these patients [18]. Our study showed that the dietary supplement Fortasyn Connect significantly improved BPSD for at least one year. Both MCI and dementia patients benefited from Fortasyn Connect, with pronounced reductions in NPI total scores already after 3 months, independently of diagnosis, although a stabilization of these improvements was more evident in MCI patients at month 6 and 12. This finding is in line with results from clinical trials, where benefits of Fortasyn Connect were mainly observed at the early end of the AD spectrum [19, 30–32]. Dietary supplementation combined with individualized modifications in other lifestyle aspects (such as physical exercise, cognitive training, sleep, or vascular risk control) increases the likelihood of delaying cognitive decline [33, 34]. Studies assessing the benefits of Fortasyn Connect combined with other lifestyle interventions on neuropsychiatric and cognitive symptoms are warranted.

The effects of Fortasyn Connect on synaptic activity after 3 and 6 months have been observed in prior studies that used electroencephalography (EEG) to assess synaptic activity [30, 35]. EEG changes after pharmacological treatment (galantamine or risperidone) of neuropsychiatric symptoms in dementia have been recently evaluated, and correlations between NPI agitation and delta frequencies were found at baseline and after 3 months [36]. Future studies aimed at determining the correlation between decreases in the NPI and EEG parameters would help to understand the underlying mechanisms of BPSD improvements when receiving Fortasyn Connect.

Symptoms of depression (assessed both by the NPI and GDS), anxiety, apathy, and irritability (assessed by the NPI) were significantly lower at each monitoring visit while receiving Fortasyn Connect. These improvements are of great interest for clinicians, patients, and their families. Furthermore, BPSD represent a risk factor for cognitive and dementia progression [5] so that interventions targeted to treat these symptoms may delay disease progression and are already highly recommended in MCI patients [37]. In addition, both patients and their caregivers experience significant distress and poor quality of life due to BPSD [7, 38], leading to early institutionalization of patients [39]. Depression, in particular, has been shown to increase the risk of institutionalization and even of mortality [40]. In our study, caregiver distress was significantly reduced after initiating Fortasyn Connect at each visit, providing evidence of the benefits of this dietary supplement in decreasing the disease burden of the family too.

Everyday function remained stable overall. However, results from the BLS-D and the RDRS-2 subscales were slightly different. Scores in the ADL subscale of the BLS-D showed improvement at month 3 and 6 but were identical to baseline at month 12. By contrast, scores in the ADL subscale of the RDRS-2 remained stable at month 3 and 6 but got worse at month 12. The difference between these results may lie in the specific aspects evaluated by each scale. The ADL subscale of the BLS-D focusses more on memory and orientation domains, whereas the ADL subscale of the RDRS-2 assesses mobility and coordination (for instance, eating, dressing, or grooming). The aspects evaluated by the RDRS-2 are usually impaired at later stages of the disease and therefore this tool might have been less sensitive for evaluating changes in everyday function in our cohort, where most patients had MCI. Indeed, the average RDRS-2 score during the study period (≤23.5) indicates that our patients had minimal disabilities (typical scores for elderly community residents with minimal disabilities average between 21–22). Another study that measured IADL with the Amsterdam IADL questionnaire observed that patients with mild AD receiving Fortasyn Connect had less decline in IADL after 6 and 12 months than a reference population [41]. Both studies, despite evaluating functioning with different assessment tests, suggest that Fortasyn Connect might help delay function worsening in these patients.

Despite the greater improvements observed in patients who were not receiving AChEi, NPI scores were very similar in all patients up to month 6, and only at month 12 were NPI scores in patients receiving AChEi over five points higher than in those without AChEi. Similar improvements after 6 months regardless of AChEi here apparently contrast with results from Viñuela et al. [42] who showed a synergistic effect of the combination of Fortasyn Connect and AChEi in patients with mild AD. However, Viñuela et al. assessed cognitive and functional performance (using the Clinical Dementia Rating) but did not evaluate BPSD. Taken together, these findings suggest that the effect of combining Fortasyn Connect and AChEi might have a different impact on cognitive and neuropsychiatric symptoms. In both cases, nevertheless, patients treated with AChEi also benefited from Fortasyn Connect.

Fortasyn Connect has shown a favorable safety profile [43, 44], and no tolerability or health concerns have emerged. Here, only four patients (out of 236) reported a mild gastrointestinal adverse event, confirming the good safety profile of Fortasyn Connect. The most common reason for discontinuation, however, was the price of the product, which might constitute a financial burden for families with lower incomes.

### Limitations

Due to the retrospective nature of the study and the fact that patients attended monitoring visits as per clinical practice, not all patients with data at baseline had data at each of the following visits. Even so, the number of patients with available data up to month 12 was considerably large and allowed for adequately addressing the objectives of the study. Another limitation was that modifiable lifestyle factors such as patient’s diet, physical exercise, cognitive training, or sleep that could have influenced BPSD were not assessed, and therefore their role on the benefits observed in these patients cannot be discarded. Additionally, the number of patients with dementia of etiologies different to AD was small, and therefore conclusions on the effect of Fortasyn Connect in these patients should be taken with caution. Lastly, brain mechanisms underlying changes in neuropsychiatric symptoms were unexplored. Future studies could provide further information on the benefit of Fortasyn Connect on neuropsychiatric symptoms by assessing its effect on brain function or structure in MCI and dementia patients.

### Conclusions

Fortasyn Connect improved behavior over 12 months in patients with MCI and dementia. The benefit was greater if prescribed early and if the baseline NPI score was higher. Depression, anxiety, apathy, and irritability were the symptoms that improved the most. The benefits were observed when Fortasyn Connect was used alone or combined with AChEi, antidepressants, or ginkgo biloba. Fortasyn Connect may be useful in patients with MCI and dementia of AD type or other etiology.

## Supplementary Material

Supplementary Material

## Data Availability

The data supporting the findings of this study are available on request from the corresponding author. The data are not publicly available due to privacy or ethical restrictions.
