# Supplementary material for: Fortasyn Connect Improves Neuropsychiatric Symptoms in Patients with Mild Cognitive Impairment and Dementia: Results from a Retrospective Real-World Study
Source: J Alzheimers Dis. 2023 May 16;93(2):621–31. doi: 10.3233/JAD-221122 (PMC10200227; doi:10.3233/JAD-221122)
Supplement: Supplementary Material [file jad-93-jad221122-s001.pdf]

Supplementary Material

Fortasyn Connect Improves Neuropsychiatric Symptoms in Patients with Mild Cognitive Impairment and Dementia: Results from a Retrospective Real-World Study

Supplementary Table 1. NIP scores in patients with MCI

**NPI scores**	**Baseline (n=158)**	**Month 3 (n=81)**		**Month 6 (n=109)**		**Month 12 (n=103)**	
	Mean (SD)	Mean (SD)	*p*	Mean (SD)	*p*	Mean (SD)	*p*
Number of items	3.5 (2.1)	2.2 (1.9)	<0.001*	2.2 (1.9)	0.001*	2.2 (1.8)	<0.001*
Severity	4.6 (3.3)	2.7 (2.8)	<0.001*	2.7 (2.4)	<0.001*	2.7 (2.5)	<0.001*
Caregiver distress	9.3 (6.4)	5.1 (5.6)	<0.001*	5.3 (5.2)	<0.001*	5.3 (5.0)	<0.001*
Total (severity and frequency)	15.7 (11.8)	8.5 (9.3)	<0.001*	8.2 (8.2)	<0.001*	8.5 (9.1)	<0.001*
Per item							
Delusions	0.9 (2.3)	0.4 (1.7)	0.256	0.4 (1.6)	0.071	0.2 (0.9)	<0.001*
Hallucinations	0.3 (1.1)	0.1 (0.4)	0.094	0.1 (0.5)	0.070	0.1 (0.7)	0.605
Agitation	0.5 (1.4)	0.3 (1.4)	0.096	0.1 (0.5)	0.057	0.3 (1.2)	0.661
Depression	2.5 (3.0)	1.0 (2.1)	<0.002*	1.4 (2.6)	<0.001*	1.5 (2.7)	<0.001*
Anxiety	3.0 (3.2)	1.4 (2.3)	<0.001*	1.3 (2.0)	<0.001*	1.4 (2.5)	<0.001*
Euphoria	0.1 (0.7)	0.1 (0.7)	0.458	0.1 (0.4)	0.450	0.1 (0.9)	0.888
Apathy	3.4 (3.0)	2.0 (2.7)	0.005*	2.2 (2.7)	<0.001*	2.1 (2.8)	<0.001*
Disinhibition	0.5 (1.5)	0.2 (0.8)	0.123	0.0 (0.3)	0.002*	0.1 (0.7)	0.005*
Irritability	1.9 (2.4)	1.2 (2.0)	0.039*	0.9 (1.3)	<0.001*	1.1 (2.0)	0.017*
Psychomotor alterations	0.4 (1.6)	0.1 (1.0)	>0.999	0.1 (0.9)	0.116	0.1 (0.5)	0.027*
Sleep disorders	1.4 (2.2)	0.7 (1.4)	0.126	1.0 (1.8)	0.186	1.0 (1.7)	0.492
Eating disorders	0.9 (1.7)	0.9 (1.8)	0.864	0.7 (1.5)	0.695	0.5 (1.4)	0.098

p values from comparisons between each month (month 3, month 6, and month 12) versus baseline are showed (Wilcoxon test)

Supplementary Table 2. NIP scores in patients with dementia

NPI scores	Baseline (n=78)[1]	Month 3 (n=39)		Month 6 (n=48)		Month 12 (n=44)[1]	
	Mean (SD)	Mean (SD)	*p*	Mean (SD)	*p*	Mean (SD)	*p*
Number of items	4.2 (2.2)	3.0 (2.5)	<0.002*	3.0 (2.2)	0.003*	3.3 (2.5)	0.028*
Severity	6.4 (4.1)	4.3 (4.1)	<0.001*	4.3 (4.0)	<0.001*	4.8 (4.6)	0.011*
Caregiver distress	12.0 (7.3)	6.9 (6.4)	<0.001*	8.4 (7.6)	0.001*	12.0 (9.4)	0.030*
Total (severity and frequency)	22.2 (15.2)	14.2 (13.6)	<0.001*	15.2 (15.2)	0.006*	16.2 (16.2)	0.009*
Per item							
Delusions	1.8 (3.2)	1.3 (2.5)	0.032*	1.8 (3.2)	0.878	2.0 (3.3)	0.656
Hallucinations	0.8 (2.4)	0.4 (1.4)	0.081	0.8 (2.4)	0.505	0.7 (1.9)	0.782
Agitation	0.8 (2.1)	0.4 (1.0)	0.323	0.5 (2.0)	0.482	0.5 (1.7)	0.007*
Depression	3.0 (3.5)	2.0 (3.0)	0.049*	1.7 (2.8)	0.004*	1.7 (2.6)	0.240
Anxiety	3.2 (3.7)	1.6 (2.7)	0.012*	2.3 (3.5)	0.104	1.1 (2.1)	<0.001*
Euphoria	0.3 (1.4)	0.3 (1.4)	0.999	0.2 (1.2)	0.276	0.1 (0.5)	0.285
Apathy	4.6 (3.4)	3.7 (3.8)	0.060	3.2 (3.3)	0.011*	3.4 (3.1)	0.131
Disinhibition	0.4 (1.2)	0.6 (2.0)	0.123	0.2 (0.7)	0.389	0.2 (0.8)	0.570
Irritability	2.2 (2.8)	1.3 (2.2)	0.014*	1.5 (2.3)	0.171	1.8 (2.4)	0.191
Psychomotor alterations	1.2 (2.7)	0.5 (1.6)	0.100	0.6 (1.8)	0.370	0.7 (2.5)	0.440
Sleep disorders	1.7 (3.0)	0.8 (2.0)	0.138	1.0 (2.1)	0.405	1.4 (2.4)	0.069
Eating disorders	1.6 (2.4)	1.2 (2.2)	0.068	1.5 (2.4)	0.977	1.0 (2.1)	0.183

[1] Data per item was missing in one patient;

Supplementary Table 3. MMSE, GDS, BLS-D, and RDRS-2 at each visit in patients with MCI

Scores	Baseline	Month 3		Month 6		Month 12	
	Mean (SD) [n]	Mean (SD) [n]	*p*	Mean (SD) [n]	*p*	Mean (SD) [n]	*p*
MMSE	25.3 (3.3) [158]	25.5 (3.2) [84]	0.064	25.7 (3.4) [113]	0.335	25.3 (3.4) [106]	0.824
GDS	9.4 (5.6) [143]	9.2 (5.8) [36]	0.014*	8.4 (5.3) [36]	0.004*	8.4 (5.1) [47]	0.089
BLS-D	3.7 (2.3) [154]	2.6 (2.2) [81]	<0.001*	2.8 (2.4) [103]	<0.001*	3.0 (2.3) [98]	0.080
ADL	1.2 (0.8) [154]	0.9 (0.6) [81]	<0.001*	1.0 (0.9) [103]	0.036*	1.2 (1.0) [98]	0.549
Habits	0.4 (0.7) [154]	0.4 (0.7) [81]	>0.999	0.4 (0.7) [103]	0.862	0.4 (0.7) [98]	0.611
RDRS-2	21.1 (3.2) [158]	20.4 (2.9) [81]	0.040*	20.6 (2.8) [108]	0.187	21.3 (3.2) [103]	0.740
ADL	9.8 (1.9) [158]	8.7 (1.4) [81]	0.325	8.8 (1.4) [108]	0.920	9.1 (1.9) [103]	0.224
Disability	8.5 (1.4) [158]	8.3 (1.5) [81]	0.235	8.4 (1.6) [108]	0.425	8.6 (1.6) [103]	0.432

Supplementary Table 4. MMSE, GDS, BLS-D, and RDRS-2 at each visit in patients with dementia

Scores	Baseline	Month 3		Month 6		Month 12	
	Mean (SD) [n]	Mean (SD) [n]	*p*	Mean (SD) [n]	*p*	Mean (SD) [n]	*p*
MMSE	21.4 (3.7) [78]	22.1 (4.0) [41]	0.002*	22.1 (3.6) [48]	0.672	20.5 (4.7) [45]	0.150
GDS	9.4 (6.4) [67]	4.8 (5.2) [14]	0.003*	7.8 (5.0) [16]	0.505	6.5 (4.7) [16]	0.196
BLS-D	6.9 (3.7) [76]	6.2 (4.2) [37]	0.018*	5.3 (3.7) [46]	0.009*	6.9 (4.2) [41]	0.426
ADL	2.6 (1.6) [76]	2.4 (1.7) [37]	0.047*	2.2 (1.3) [46]	0.113	2.9 (1.9) [41]	0.118
Habits	1.1 (1.5) [76]	1.4 (1.6) [37]	0.831	1.1 (1.6) [46]	0.970	1.6 (1.7) [41]	0.009
RDRS-2	26.4 (6.0) [77]	26.5 (7.2) [39]	0.055	25.8 (6.7) [47]	0.533	28.9 (7.7) [41]	0.740
ADL	11.7 (4.3) [77]	12.6 (5.6) [39]	0.491	11.8 (4.7) [47]	0.789	13.8 (5.7) [41]	0.004*
Disability	10.5 (1.9) [77]	10.1 (1.8) [39]	0.001*	10.0 (2.0) [47]	0.259	11.0 (2.4) [41]	0.227
